# Supplementary material for: Simultaneous orientation and 3D localization microscopy with a Vortex point spread function
Source: Nat Commun. 2021 Oct 11;12:5934. doi: 10.1038/s41467-021-26228-5 (PMC8505439; doi:10.1038/s41467-021-26228-5)
Supplement: Supplementary file 1 — Supplementary Information [file 41467_2021_26228_MOESM1_ESM.pdf]

Simultaneous orientation and 3D localization microscopy with a Vortex point spread function
Supplementary Information

Christiaan N. Hulleman[1,2], Rasmus Ø. Thorsen[1,2], Eugene Kim[3,4], Cees Dekker[3], Sjoerd Stallinga[1,2,*], and Bernd Rieger[1,2,*]

[1]Department of Imaging Physics, Delft University of Technology, Delft, The Netherlands
[2]These authors contributed equally: Christiaan N. Hulleman, Rasmus Ø. Thorsen, Sjoerd Stallinga and Bernd Rieger
[3]Department of Bionanoscience, Kavli Institute of Nanoscience, Delft University of Technology, Delft, The Netherlands
[4]Max Planck Institute of Biophysics, 60438 Frankfurt, Germany
[*]e-mail: s.stallinga@tudelft.nl, b.rieger@tudelft.nl

Contents

1 Supplementary Note 1: Imaging PSF model **3**

2 Supplementary Note 2: Field dependent aberration coefficients **5**

List of Tables

1 Localization density 7
2 Plectoneme localization density 8
3 Theoretical precision limit (CRLB) comparison 8

List of Figures

1 Simulated Vortex PSF performance 9
2 Performance as a function of signal to background ratio 10
3 Cramér–Rao bounds versus axial position 11
4 Importance of calibrated aberrations 12
5 Aberration map 13
6 Normal PSF z-stack compared to single-slice Vortex PSF 14
7 Influence of cover slip refractive index on localization bias 15
8 Influence of aberration map on λ-DNA FWHM 16
9 λ-DNA z surface 17
10 Experimental precision of λ-DNA data 18
11 Orientation distributions of various λ-DNA strands 19
12 Orientation distribution of fixed molecules 20
13 Spatial autocorrelation of additional plectonemes 21
14 Spatial autocorrelation of torsionally relaxed DNA molecules . . 22
15 Spatial autocorrelation of λ-DNA 23
16 Orientation distribution of the supercoil and plectoneme 24
17 Optical setup 25
18 Vortex phase plate alignment 26
19 Fine drift correction 27

1 Supplementary Note 1: Imaging PSF model

We use a full vectorial PSF model in the Vortex PSF fitting, as initially described in [1], and extend it to incorporate varying degrees of orientational constraint and orientational diffusion [2]. The expected photon count at pixel l depends on the molecule position $\boldsymbol{r}_0 = (x_0, y_0, z_0)$, the signal photon count N, background photons per pixel b, and the molecular orientation $\Omega_0 = (\phi_0, \theta_0)$ together with the degree of orientational constraint g_2, giving a total of 8 parameters. The integration of the model H gives the expected photon count:

$$\mu_l = \int_{D_l} \mathrm{d}x\mathrm{d}y \, H(\boldsymbol{r} - \boldsymbol{r}_0, \Omega) \tag{1}$$

where the integration is over the pixel area D_l of size $a \times a$. The image formation model is taken to be the weighted sum of the free dipole PSF and the orientation-dependent fixed dipole PSF, where the relative weights are determined by g_2, plus a constant background. There are more elaborate rotational diffusion models [3, 4, 5, 6] however this model is appropriate and sufficient for rotational diffusion with a rotational relaxation time faster than the fluorescence lifetime [2], independent of the type of rotational constraint. This model can be written as

$$H(\boldsymbol{r}, \Omega) = N \left[\frac{(1 - g_2)}{3} H_{\mathrm{free}}(\boldsymbol{r}) + \frac{g_2}{3} H_{\mathrm{fixed}}(\boldsymbol{r}, \Omega) \right] + \frac{b}{a^2} \tag{2}$$

The PSF of a fixed dipole emitter is

$$H_{\mathrm{fixed}}(\boldsymbol{r}, \Omega) = \sum_{h,j=x,y,z} A_{hj}(\boldsymbol{r}) d_h(\Omega) d_j(\Omega) \tag{3}$$

where $d_h(\Omega)$ are the components of the dipole unit vector

$$\boldsymbol{d}(\Omega) = (\sin\theta \cos\phi, \sin\theta \sin\phi, \cos\theta). \tag{4}$$

The average PSF of a freely rotating dipole emitter is

$$H_{\mathrm{free}}(\boldsymbol{r}) = \frac{1}{3} \sum_{h=x,y,z} A_{hh}(\boldsymbol{r}) \tag{5}$$

with

$$A_{hj}(\boldsymbol{r}) = \sum_{k=x,y} w_{kh}(\boldsymbol{r}) w^*_{kj}(\boldsymbol{r}) \tag{6}$$

where the functions $w(\boldsymbol{r})$ represent the electric field component in the image plane proportional to the emission dipole component $j = x, y, z$. These functions can be expressed as integrals over the pupil plane:

$$w_{kj}(\boldsymbol{r}) = \frac{1}{\pi} \int \mathrm{d}^2\rho \, A(\boldsymbol{\rho}) \exp\left(\frac{2\pi i W(\boldsymbol{\rho})}{\lambda} \right) q_{kj}(\boldsymbol{\rho}) \exp(-i\boldsymbol{k} \cdot \boldsymbol{r}) \tag{7}$$

where the integration is over normalized pupil coordinates $\boldsymbol{\rho}$, $A(\boldsymbol{\rho})$ is the aplanatic amplitude correction factor, and $q_{kj}(\boldsymbol{\rho})$ are the polarization vectors given in full detail in [7]. The wavevector $\boldsymbol{k}(\boldsymbol{\rho})$ depends on the normalized pupil coordinates by

$$\boldsymbol{k}(\rho) = \frac{2\pi}{\lambda}\left(\mathrm{NA}\rho_x, \mathrm{NA}\rho_y, \sqrt{n^2 - \mathrm{NA}^2\boldsymbol{\rho}}\right) \tag{8}$$

with n the refractive index of the medium.

The aberration function $W(\boldsymbol{\rho}) = K(\boldsymbol{\rho}) + W_{\mathrm{abb}}(\boldsymbol{\rho})$ includes the zone function of the vortex phase plate: $K(\boldsymbol{\rho}) = \beta/(2\pi)$ where $\beta = \arctan(\rho_x/\rho_y)$ is the azimuth pupil coordinate, and further includes field-dependent aberrations $W_{\mathrm{abb}}(\boldsymbol{\rho})$ as described in Supplementary Note 2.

The imaging model's partial derivatives with respect to the parameters are needed for the MLE optimization routine. These are easy to evaluate for the signal photon count N, background photons per pixel b, and diffusion weights g_2 as these appear linearly in the imaging model μ_k. The derivatives with respect to the fit parameters $\Theta = (x, y, z, \phi, \theta)$ are similar to [8] but now slightly more elaborate:

$$\begin{aligned}\frac{\partial \mu_l}{\partial \Theta} = N \Bigg[&\frac{1-g_2}{3} \sum_{k=x,y} \sum_{j=x,y,z} \int \mathrm{d}^2\rho \, 2\Re\left\{ w_{kj}^* \frac{\partial w_{kj}}{\partial \Theta} \right\} \\ &+ \frac{g_2}{3} \sum_{k=x,y} \sum_{j=x,y,z} \int \mathrm{d}^2\rho \, 2\Re\left\{ w_{kj}^* d_j \frac{\partial \left(w_{kj} d_j\right)}{\partial \Theta} \right\} \Bigg]\end{aligned} \tag{9}$$

where $w_{kj} = w_{kj}(r - r_0)$ and $d_j = d_j(\Omega)$. The derivatives of w_{kj} with respect to the coordinates of the emitter are needed here:

$$\begin{aligned}\frac{\partial w_{kj}(\boldsymbol{r} - \boldsymbol{r}_0)}{\partial \boldsymbol{r}_0} = &\frac{i}{\pi} \int \mathrm{d}^2\rho \, A(\boldsymbol{\rho}) \exp\left(\frac{2\pi i W(\boldsymbol{\rho})}{\lambda}\right) q_{kj}(\boldsymbol{\rho}) \\ &\times \boldsymbol{k}(\boldsymbol{\rho}) \exp(-i\boldsymbol{k}(\boldsymbol{\rho}) \cdot (\boldsymbol{r} - \boldsymbol{r}_0))\end{aligned} \tag{10}$$

as are the derivatives of the dipole vector $\boldsymbol{d}(\Omega)$ with respect to the polar and azimuthal angles:

$$\frac{\partial \boldsymbol{d}(\Omega)}{\partial \phi} = (-\sin\theta \sin\phi, \sin\theta \cos\phi, 0) \tag{11}$$

$$\frac{\partial \boldsymbol{d}(\Omega)}{\partial \theta} = (\cos\theta \cos\phi, \cos\theta \sin\phi, -\sin\theta). \tag{12}$$

2 Supplementary Note 2: Field dependent aberration coefficients

The aberrations function $W_{\mathrm{abb}}(\boldsymbol{\rho})$ is conventionally expressed as a linear sum of the root mean square (RMS) normalized Zernike polynomials $Z_n^m(\boldsymbol{\rho})$: $W_{\mathrm{abb}}(\boldsymbol{\rho}) = \sum_{n,m} A_n^m Z_n^m(\boldsymbol{\rho})$. In most studies the Zernike coefficients are constant parameters. In this study we follow the approach of [9] and [10] by taking the dependence on the position in the FOV into account, i.e. we take the Zernike coefficients to be functions of the field coordinates (x, y). These functions $A_n^m(x, y)$ are determined from a calibration procedure. We make a through-focus image stack of a set of beads randomly spread over the FOV, as opposed to the procedure of [10], where a single bead is positioned on a series of grid positions by the microscope stage. For each bead the Zernike coefficients are retrieved using our previous method [11]. According to Nodal Aberration Theory (NAT), the aberration coefficients $A_n^m(x, y)$ can be suitably described by low order Taylor series in x and y:

$$A_n^m(x, y) = \sum_{jk} \gamma_{nmjk} x^j y^k. \tag{13}$$

The set of coefficients γ_{nmjk} of these Taylor series for different positions are related [12], which we use to our advantage as this decreases the number of parameters to be determined from experiments. The NAT-model is fitted to the measured A_n^m at the beads' positions by a straightforward least-squares fit. With this calibration procedure, the estimated Zernike coefficients can effectively be interpolated over the entire imaging field.

We take into account Zernike modes with $n+|m| \leq 6$, which include primary and secondary astigmatism, coma, spherical aberration, and trefoil, and use the analysis of [13], where polynomials in the field coordinates up to order $6-n$ are used in the NAT description of the field dependence of the contributing Zernike modes.

In the following, these expressions are summarized as implemented in our fitting using a set of perturbation coefficients $(\chi, \xi, \delta, \mu, \eta, \kappa, \nu)$. Primary astigmatism with perturbation coefficients χ is given by:

$$\begin{aligned} A_2^{-2} = {} & \chi_1(x^3y + xy^3) + \chi_2(x^2y + y^3) + \chi_3(x^3 + xy^2) \\ & + \chi_4(x^2 + y^2) + \chi_6(xy^2 - x^3) + \chi_7 2xy^2 \\ & + \chi_8 y - \chi_9 x + \chi_{10}(2xy^2) + \chi_{11} x + \chi_{12} y + \chi_{13} \end{aligned} \tag{14}$$

$$\begin{aligned} A_2^{2} = {} & \chi_1(y^4 - x^4) - \chi_2(x^3 + xy^2) + \chi_3(x^2y + y^3) \\ & + \chi_5(x^2 + y^2) - \chi_6 2x^2y + \chi_7(y^3 - x^2y) \\ & + \chi_8 x + \chi_9 y + \chi_{10}(y^2 - x^2) + \chi_{11} y - \chi_{12} x + \chi_{14}, \end{aligned} \tag{15}$$

primary coma with coefficients ξ by:

$$
\begin{aligned}
A_3^{-1} &= \xi_1(x^3 + xy^2) + \xi_2 x^2 + \xi_3 xy + \xi_4 x + \xi_5(x^2 + y^2) \\
&\quad + \xi_7 y + \xi_8 x + \xi_9 \qquad (16) \\
A_3^{1} &= \xi_1(y^3 + x^2 y) + \xi_2 xy + \xi_3 y^2 + \xi_4 y + \xi_6(x^2 + y^2) \\
&\quad + \xi_7 x - \xi_8 y + \xi_{10} \qquad (17)
\end{aligned}
$$

and primary spherical aberration with coefficients δ by:

$$A_4^0 = \delta_1(x^2 + y^2) + \delta_2 x + \delta_3 y + \delta_4. \qquad (18)$$

The next aberration order, trefoil with coefficients μ is given by:

$$A_3^{-3} = \mu_1(3y^2 x - x^3) + \mu_2(y^2 - x^2) + \mu_3 2xy + \mu_4 x + \mu_5 y + \mu_6 \qquad (19)$$

$$A_3^{3} = \mu_1(y^3 - 3x^2 y) - \mu_2 2xy + \mu_3(y^2 - x^2) + \mu_4 y - \mu_5 x + \mu_7 \qquad (20)$$

secondary astigmatism with coefficients η by:

$$A_4^{-2} = \eta_1 2xy + \eta_2 y + \eta_3 x + \eta_4 \qquad (21)$$

$$A_4^{2} = \eta_1(y^2 - x^2) - \eta_2 x + \eta_3 y + \eta_5 \qquad (22)$$

secondary coma with coefficients κ by:

$$A_5^{-1} = \kappa_1 y + \kappa_2 \qquad (23)$$

$$A_5^{1} = \kappa_1 x + \kappa_3 \qquad (24)$$

and finally we include secondary spherical aberration, which is expected to be constant in the included polynomial order but here modeled to match equation (18), with coefficient ν:

$$A_6^0 = \nu_1(x^2 + y^2) + \nu_2 x + \nu_3 y + \nu_4. \qquad (25)$$

The independent perturbation coefficients are determined using a least-squares fit over all bead measurements to relate the perturbation coefficient to the Zernike coefficients for the field coordinates. Once the perturbation coefficients are known, the equations (14 – 25) are used as predictors based on the detected molecule position. The retrieved field-dependent Zernike aberration coefficients for our microscope are shown in Supplementary Fig. 5.

Table 1: Localizations (locs) analyzed in each figure, along with the amount of localizations in an analyzed subset, estimated length and localization density.

Figure	Total # locs	# Locs in subset	Length (μm)	Loc density (nm^{-1})[a]
Fig. 4(b-d)	6429	2375	7.02	0.34
Fig. 5(b)	4183	-	8.08	0.52
Fig. 5(c)	10409	-	7.93	1.31
Fig. 6(f)	9285	928	5.35	0.17
S. Fig. 13(b)	5646	509	3.74	0.14
S. Fig. 13(c)	7347	792	4.17	0.19
S. Fig. 14(b) $\tilde{\theta} = 148°$	11485	1294	13.18	0.10
S. Fig. 14(b) $\tilde{\theta} = 32°$	11485	1190	13.18	0.09
S. Fig. 14(c) $\tilde{\theta} = 148°$	8737	1220	7.96	0.15
S. Fig. 14(c) $\tilde{\theta} = 32°$	8737	921	7.96	0.12
S. Fig. 14(d) $\tilde{\theta} = 148°$	4306	536	5.05	0.11
S. Fig. 14(d) $\tilde{\theta} = 32°$	4306	337	5.05	0.07
S. Fig. 14(e) $\tilde{\theta} = 148°$	7425	980	7.45	0.13
S. Fig. 14(e) $\tilde{\theta} = 32°$	7425	538	7.45	0.07
S. Fig. 14(f) $\tilde{\theta} = 148°$	6950	1034	6.85	0.15
S. Fig. 14(f) $\tilde{\theta} = 32°$	6950	587	6.85	0.09
S. Fig. 14(g) $\tilde{\theta} = 148°$	5189	543	5.61	0.10
S. Fig. 14(g) $\tilde{\theta} = 32°$	5189	371	5.61	0.07
S. Fig. 15(a) $\tilde{\theta} = 148°$	9153	579	7.88	0.07
S. Fig. 15(a) $\tilde{\theta} = 32°$	9153	759	7.88	0.10
S. Fig. 15(b) $\tilde{\theta} = 148°$	9153	1336	7.88	0.17
S. Fig. 15(b) $\tilde{\theta} = 32°$	9153	1589	7.88	0.20

[a] Localization density is the ratio of localizations within the orientation subset divided by the length of the section. Unless the figure does not refer to a specific orientation subset, then it is the total number of localizations divided by the length.

Table 2: Localization (loc) density and length of different sections of the plectoneme construct. The two pre-coil parts are from the sections before overlap and intertwining itself.

Figure	Length (μm)[a]	Section	Loc density (nm^{-1})
Fig. 6(c)	1.00	pre-coil top	0.70
Fig. 6(c)	0.41	pre-coil bottom	1.13
Fig. 6(c)	5.81	coil	1.73
Total length (μm):	13.03	Density ratio:	1.89
S.Fig. 13(b)	1.54	pre-coil top	0.64
S. Fig. 13(b)	0.73	pre-coil bottom	1.02
S. Fig. 13(b)	5.01	coil	1.51
Total length (μm):	12.29	Density ratio:	1.82
S. Fig. 16(b)[b]	1.96	pre-coil left	0.80
S. Fig. 16(c)[b]	0.93	pre-coil right	0.87
S. Fig. 16(d)[b]	4.80	coil	1.76
Total length (μm):	12.49	Density ratio:	2.11

[a] The length here includes clusters at the initial DNA attachment site and where it turns around at the end of the plectoneme, this is longer than the length used for localization density analysis. For the localization density analysis, shorter uniform sections are analyzed.

[b] Same DNA molecule as Fig. 13(c).

Table 3: Theoretical precision limit (CRLB) comparison of various methods. The simulation parameters are set to match those from other methods, if unspecified our standard simulation parameters as described in the methods were used.

Method	N	b	σ_ϕ (°)	σ_θ (°)	σ_α (°)	σ_x (nm)	σ_y (nm)	σ_z (nm)
Tri-spot[a]	3,000	10	8	7	14	-	-	-
Vortex	3,000	10	8.4	2.2	23.4	2.7	3.1	-
xyPol[b]	380	2	4	15	19	10	10	-
Vortex	380	2	12.6	5.2	36.1	10.3	10.1	-
CHIDO[c]	10,000	0	0.4	0.5	6	0.85	0.95	3
Vortex	10,000	0	0.95	0.68	8.7	1.1	0.83	5.3
CHIDO[c]	10,000	250	1.1	1.6	13	2.8	2.6	9.2
Vortex	10,000	250	2.98	1.31	17	2.63	1.93	16.9

[a] Tri-spot [14], $\phi = 90°$, $\theta = 90°$, $g_2 = 1$, NA = 1.3, $n_{\text{medium}} = 1.518$, $\lambda = 600$ (nm), explicit values.

[b] xyPol (Polarized PSF) [15] $\phi = 135°$ $\theta = 90°$, $g_2 = 1$, NA = 1.4, $n_{\text{medium}} = 1.334$, $\lambda = 610$ (nm), pixelsize 58.5 (nm), values from graph.

[c] CHIDO [16], $\phi = 0°$, $\theta = 90°$, $g_2 = 1$, NA = 1.45, $\lambda = 520$ (nm), pixelsize 67 (nm), ROI=21x21, values from graph.

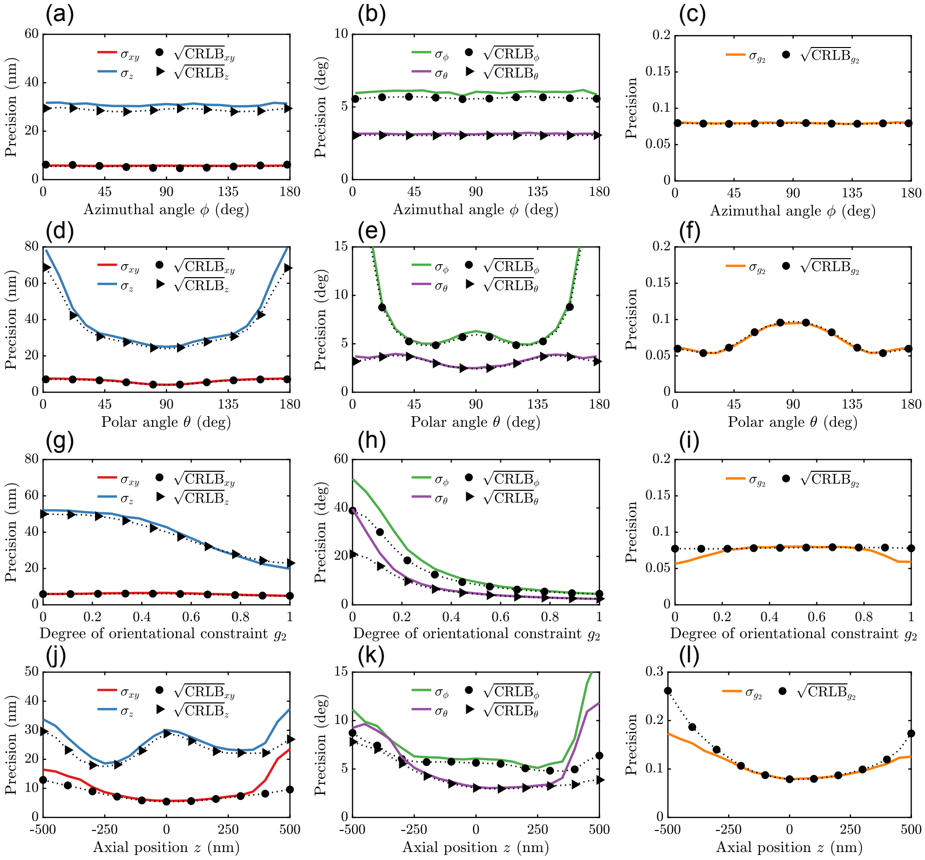

Figure 1: Simulation study of the impact of molecule orientation, rotation diffusion, and axial position on the precision. (a) Average lateral and axial localization precision, (b) orientation, and (c) g_2 precision as a function of the molecule's azimuthal angle with its polar angle uniformly chosen on a sphere with $g_2 = 0.75$. (d-f) Precision as a function of the polar angle with its azimuthal angle uniformly chosen with $g_2 = 0.75$. (g-i) Precision as a function of degree of orientational constraint g_2 with the molecule's orientation uniformly chosen on a sphere. (j-l) Precision as a function of the emitter's axial position, again with the molecule's orientation uniformly chosen on a sphere ($g_2 = 0.75$). The estimator's performance (solid colorized lines) is at the CRLB (black dashed lines with symbols) for all molecule orientations, almost regardless of the rotational diffusion. There is a slight deviation in the angles at low g_2 values (close to freely rotating dipole limit). The g_2 precision drops below the CRLB in (i,l) as the estimation converges to the boundaries $[0, 1]$. The estimator achieves the CRLB for axial positions of $|z| < 300$ nm and starts to diverge outside this region as the Vortex PSF footprint becomes too large to be contained within the ROI of 15×15 pixels. The simulation parameters are as described in the Methods.

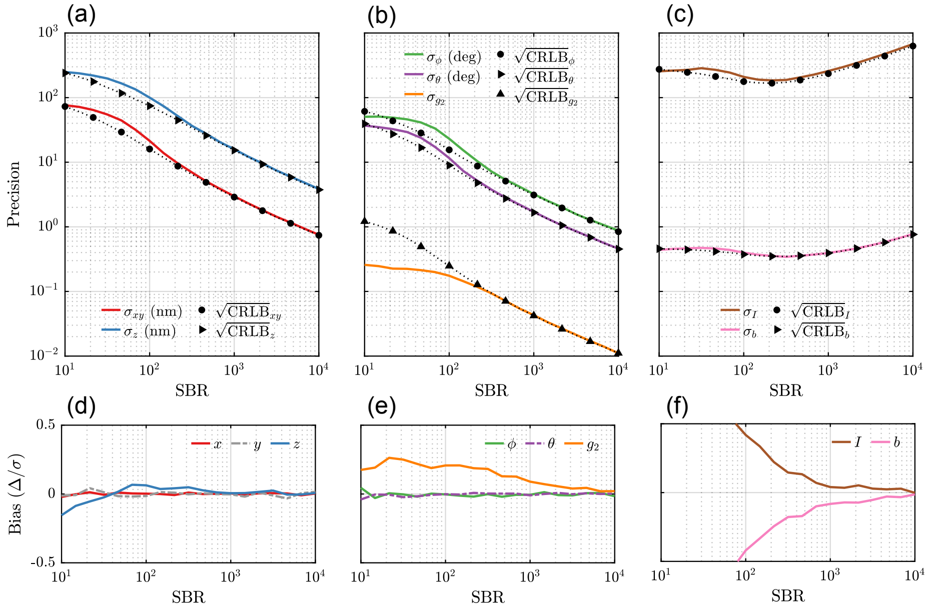

Figure 2: Simulation study of the impact of signal to background ratio (SBR = N/b) on precision and bias of the estimated parameters. (a) Average lateral and axial localization precision using a Vortex PSF model on simulated emitters, simulated with the vectorial PSF (see Methods) as a function of SBR. (b) The orientation precision of the azimuthal and polar angles together with the degree of orientational constraint. (c) The precision of the number of signal photons and of the number of background photons per pixel. The estimation performance is at the CRLB except at SBR levels below 2×10^2. (d-f) Similarly, the bias Δ normalized with the precision of the estimated parameters for the (d) position coordinates, (e) orientation parameters, and (f) photon count parameters. The simulation parameters are as described in the Methods. The slight bias in g_2 is primarily caused by the limited estimation range $[0, 1]$. At very low SBR, rotational diffusion estimates can be inaccurate due to shot noise [4].

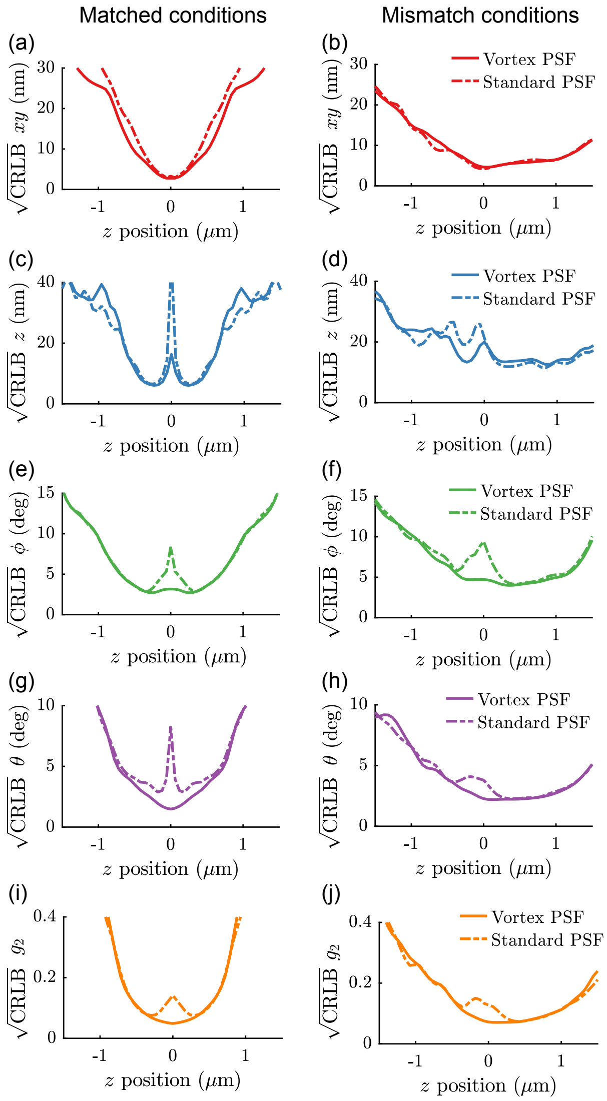

Figure 3: Simulation study of the impact of axial position on the Cramér-Rao lower bound (CRLB). (a) The lateral (xy) CRLBs for the Vortex (solid lines) and standard PSF (dashed lines) in matched conditions ($n = 1.518$) and (b) mismatch conditions ($n_{\text{med}} = 1.333$, $n_{\text{cov}} = 1.523$, $n_{\text{imm}} = 1.518$). Similarly the CRLBs for the (c-d) axial position z, (e-f) azimuth angle ϕ, (g-h) polar angle θ, and (i-j) degree of orientational constraint g_2. The data points are averaged over 10,000 orientational instances sampled uniformly on the unit sphere with the same instances used to compare the Vortex and standard PSF. The PSFs are simulated in a ROI of 31×31 pixels for which the CRLB is computed.

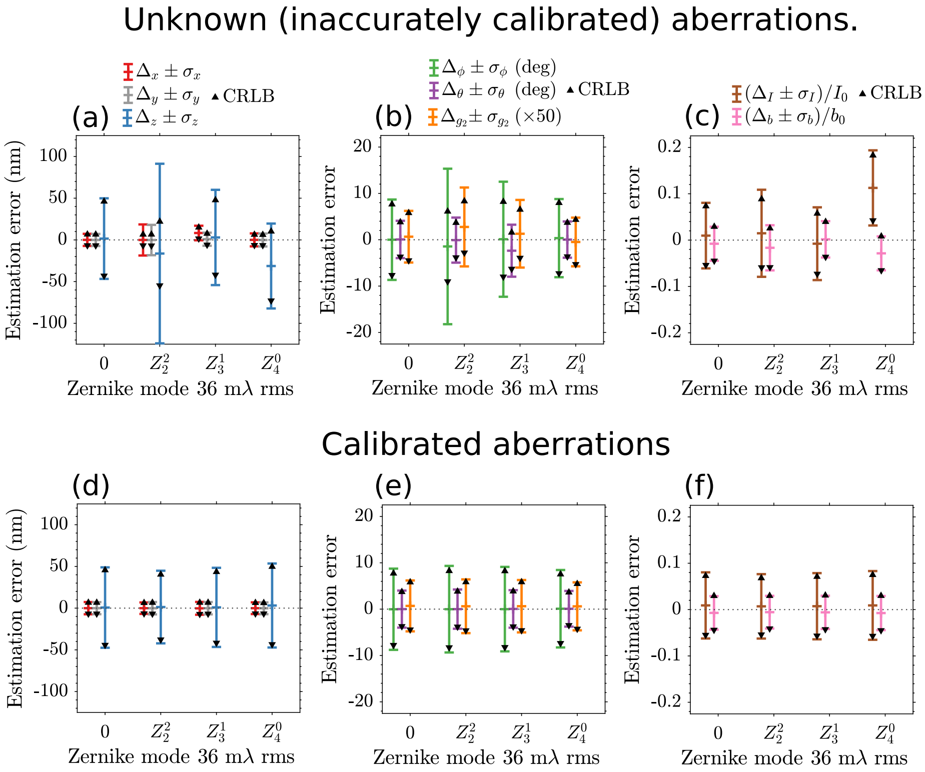

Figure 4: Simulation study of the impact of unknown and known single-mode aberrations while fitting with the Vortex PSF. (a) Lateral and axial localization error using an unaberrated Vortex PSF model on 10,000 simulated emitters with single-mode aberrations: first-order astigmatism Z_2^2, first-order coma Z_3^1, and first-order spherical Z_4^0 with RMS value of 36 mλ. The error bars indicate the mean and one standard deviation with the black marker indicating the CRLB. In the same way (b) shows the orientation error and (c) the signal and background photon error. (d-f) Same as (a-c) but including the aberrations in the Vortex PSF model. The performance is at the CRLB, and the bias is removed, when fitting with various known aberrations at 36 mλ level.

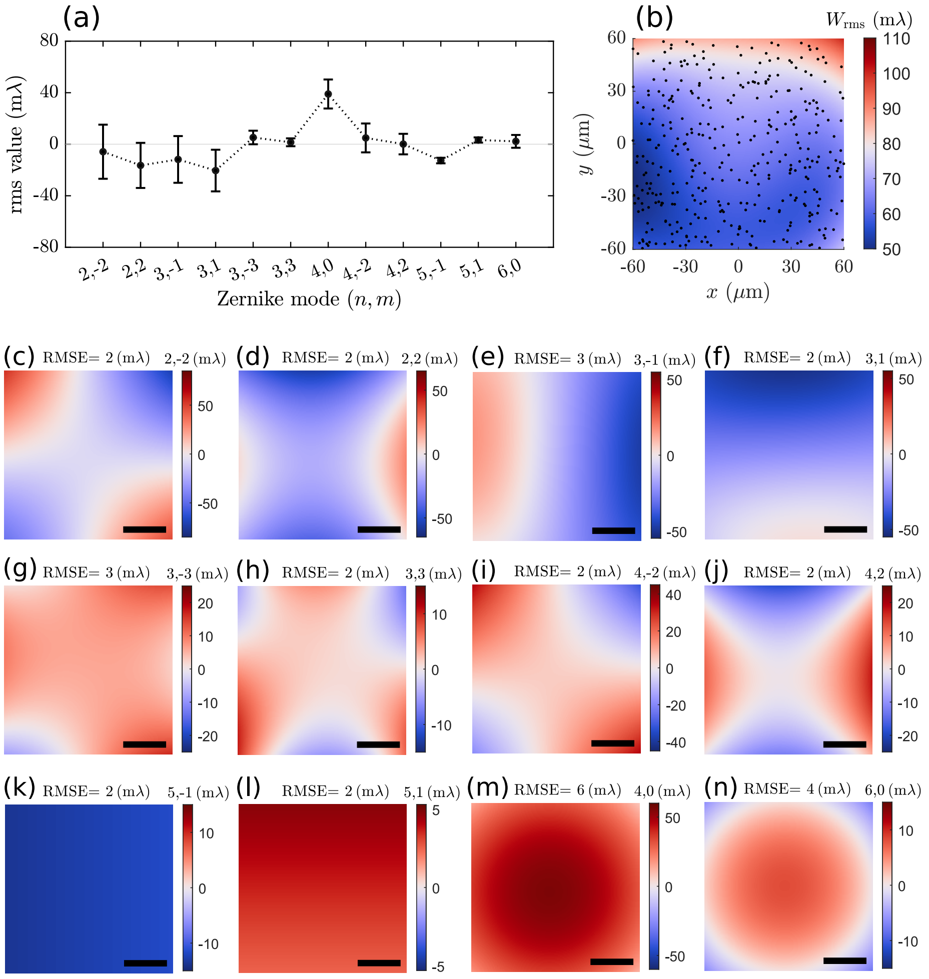

Figure 5: Quantification of aberration retrieval and correction in the field of view (FOV). (a) Fitted Zernike modes and retrieved aberration coefficients over the entire FOV. The coefficients are averaged over 429 bead localizations with error bars indicating the mean and one standard deviation. (b) The total wavefront error from the field aberration surfaces in (c-n). The black dots indicate individual bead locations. (c-n) Fitted field aberrations from the coefficients in (a) and Zernike surfaces as explained in Supplementary Note 2, with RMSE as the quality of fit. The field dependency is well-described, as the RMSE is below 6 mλ in all Zernike maps. The quality of fit is further characterized by R^2 values above 0.9 for (c, d, e, f, i, j) and above 0.5 in (g, h, m, n), and smaller when the mode appears constant in the imaged FOV (k, l). Scale bars in (c-n) are 30 μm.

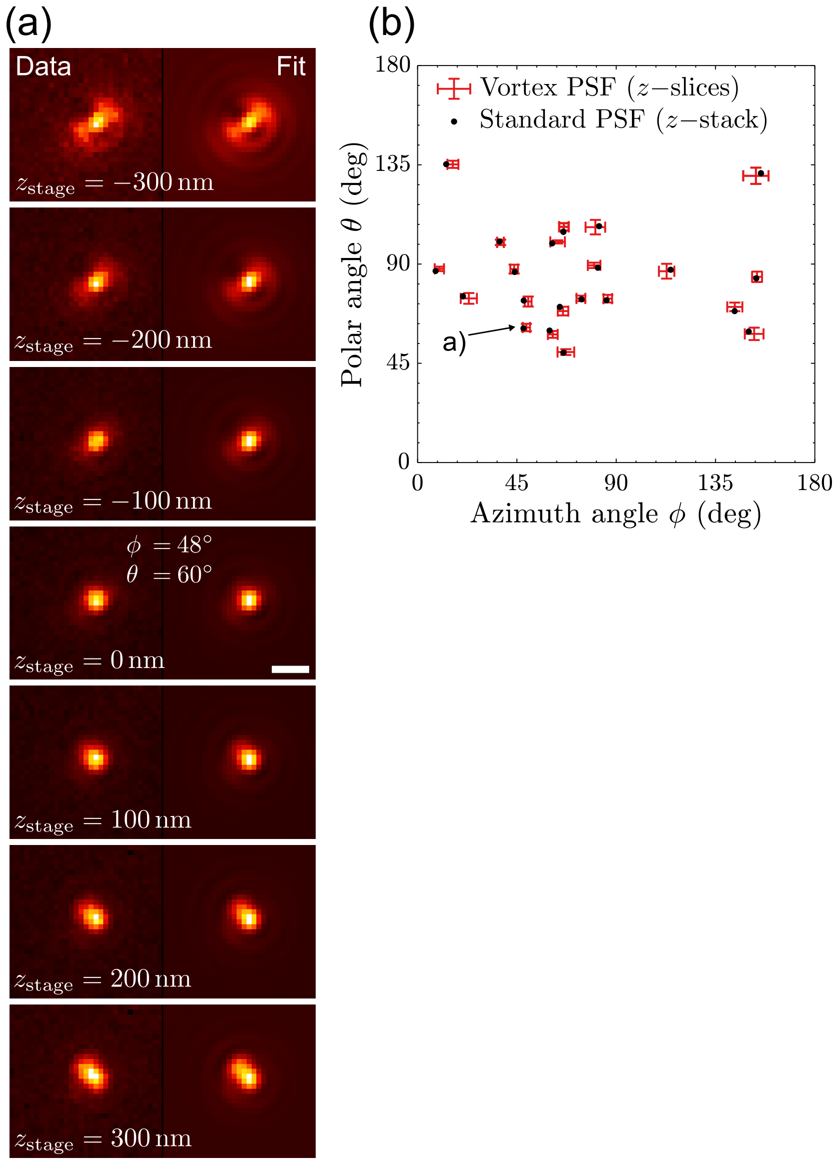

Figure 6: (a) Standard PSF model fitted to an entire z-stack resulting in the estimated orientations of $\phi = 48°$ $\theta = 60°$. This is the same molecule from Fig. 2, where it was imaged and fitted with a Vortex PSF frame by frame. (b) Estimated orientations of 21 fixed molecules from a standard PSF z-stack and single-frame Vortex PSFs with error bars indicating the mean and one standard deviation. These results indicate that the single z slice estimates with the Vortex PSF and the estimate on the entire through-focus stack with the standard PSF are mutually unbiased.

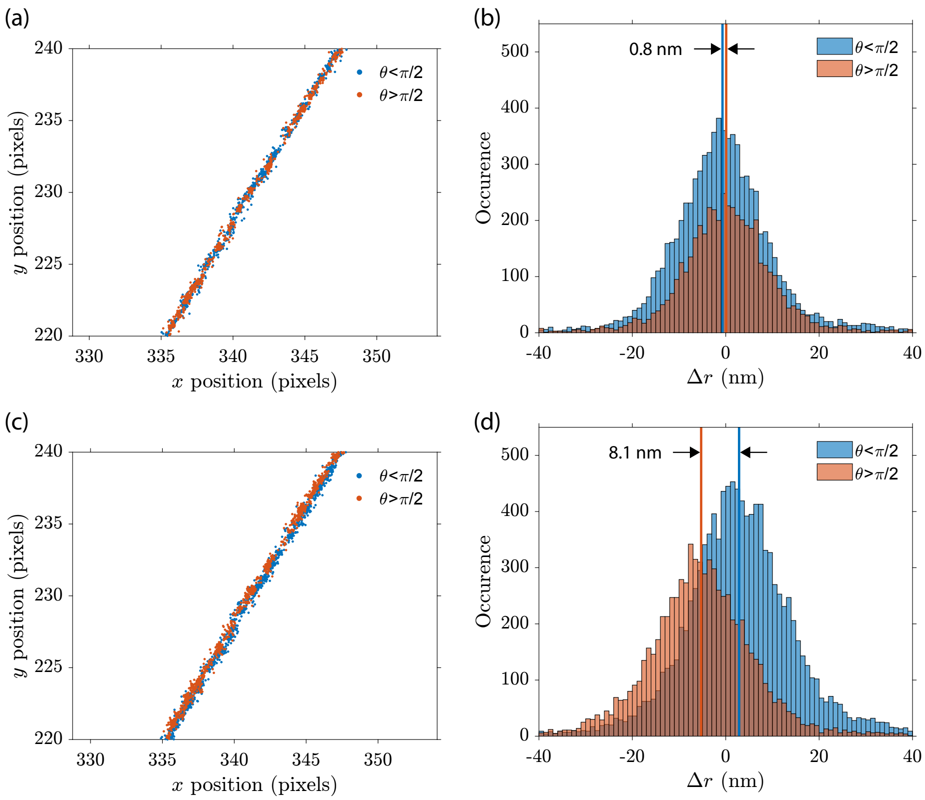

Figure 7: The influence of cover slip refractive index on localization bias. (a) Localizations of a single λ-DNA strand with an optimized cover slip refractive index of $n_{\mathrm{cov}} = 1.5209$ split into two subsets with $\theta < \pi/2$ and $\theta > \pi/2$, showing no orientation dependent shift between the two subsets. (b) Histogram of the deviations from the λ-DNA strand with a distance of 0.8 nm between the mean of the two subsets. The total width of the distribution of localizations from the strand in (a) (including both subsets) is 18.6 nm FWHM. (c) Localizations of the same λ-DNA strand fitted with a catalogue value of the cover slip refractive index of $n_{\mathrm{cov}} = 1.523$ and the same splitting into two subsets, showing a bias between the two subsets. (d) The histogram of deviations from the central λ-DNA axis highlights this shift where there is 8.1 nm between the mean position of the two subsets. This slight variation in the cover slip refractive index broadens the width of the distribution of localizations on the same λ-DNA strand from (a) to 22.8 nm FWHM.

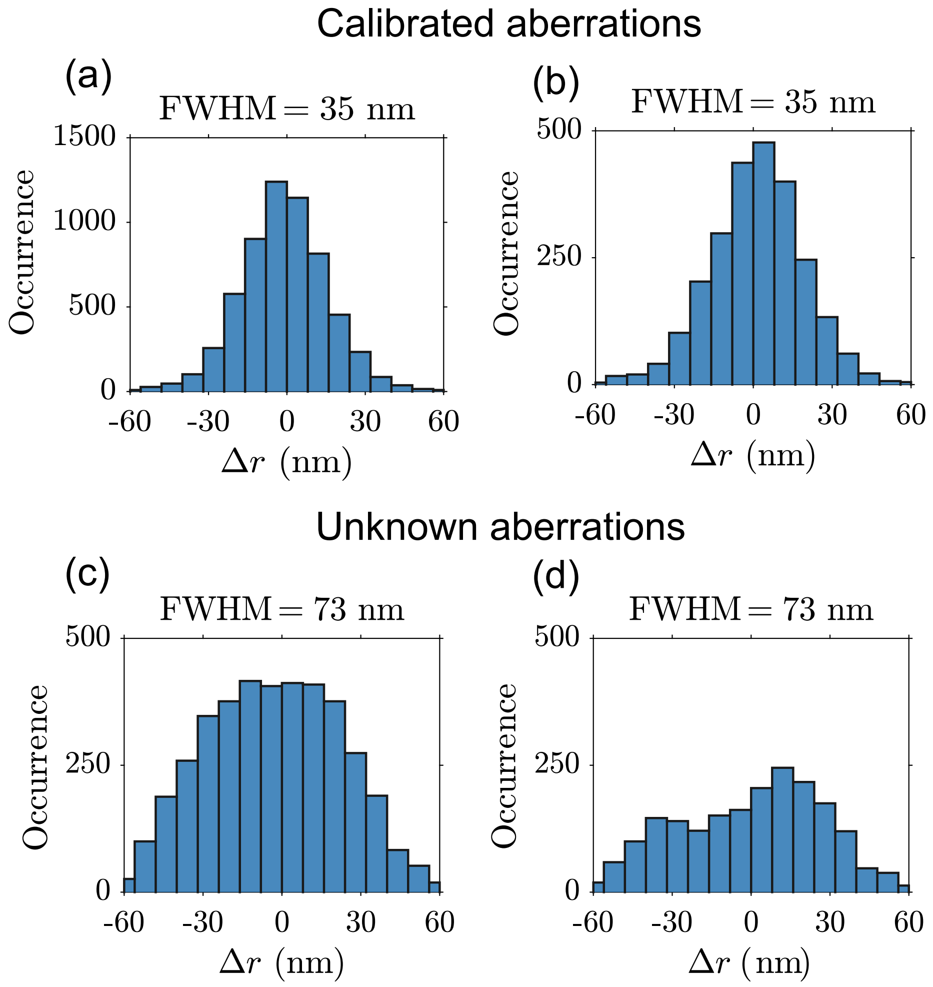

Figure 8: The effect of our calibrated PSF model for the localizations of the $\lambda-$DNA in the main text (Fig. 4(a)). (a) Position deviation from the spline fit to the DNA axis using a calibrated PSF model results in a Gaussian-like distribution with FWHM $=$ 35 nm and (b) for polar angles $45 \leq \theta \leq 135$ results in a similar distribution with FWHM $=$ 35 nm. This is wider than the value in the main text as the catalogue refractive index is used and no additional fine drift correction is applied. (c) Using a PSF model without calibrated aberrations results in a broad distribution with FWHM $=$ 73 nm and (d) for polar angles $45 \leq \theta \leq 135$ results in a non-uniform distribution with FWHM $=$ 73 nm. (a-b) The FWHM is evaluated using a normal distribution fit with support $\Delta r = \pm 40$ nm and (c-d) a non-parametric fit with a normal kernel.

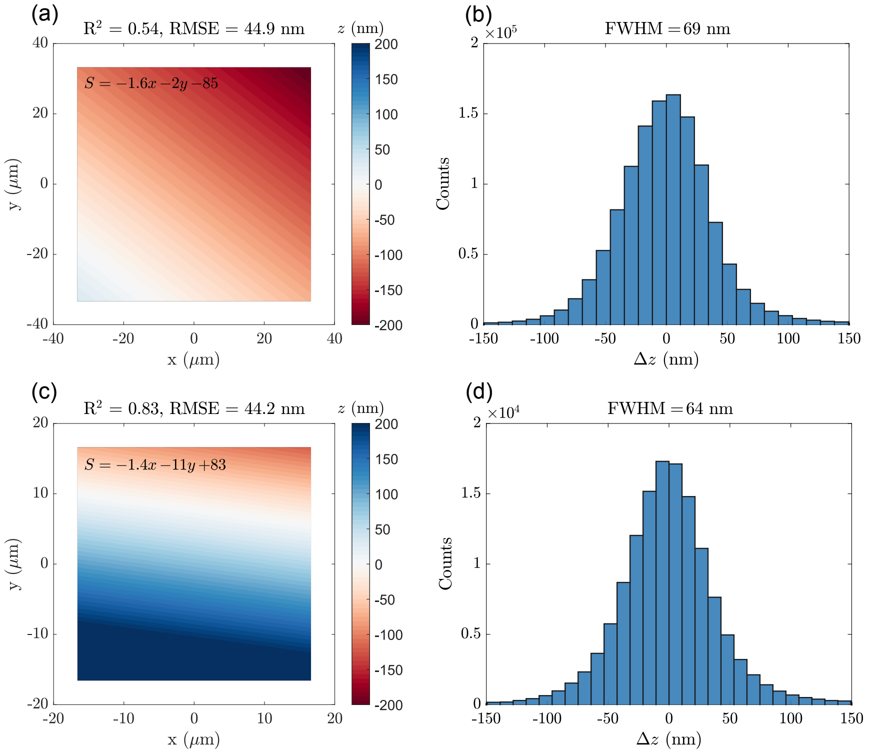

Figure 9: Planar surface fit on λ-DNA localization data, with and without an additional 0.4° sample tilt in the y direction. (a) Planar surface fit to non-tilted λ-DNA sample, where S is the surface function defining the average z position in nm as a function of the x and y position in μm. (b) Histogram of the Δz localization deviations from the surface given in (a). (c) Planar surface fit to intentionally tilted λ-DNA sample, the tilt is apparent from the larger coefficient of the y-coordinate in S compared to the coefficient in the fitted plane in (a). (d) Histogram of the Δz localization deviations from the surface given in (c). Localizations are filtered with $g_2 \geq 0.6$ to keep the localizations that bind to the lambda-DNA, thereby ensuring a robust z-estimation of the sample plane.

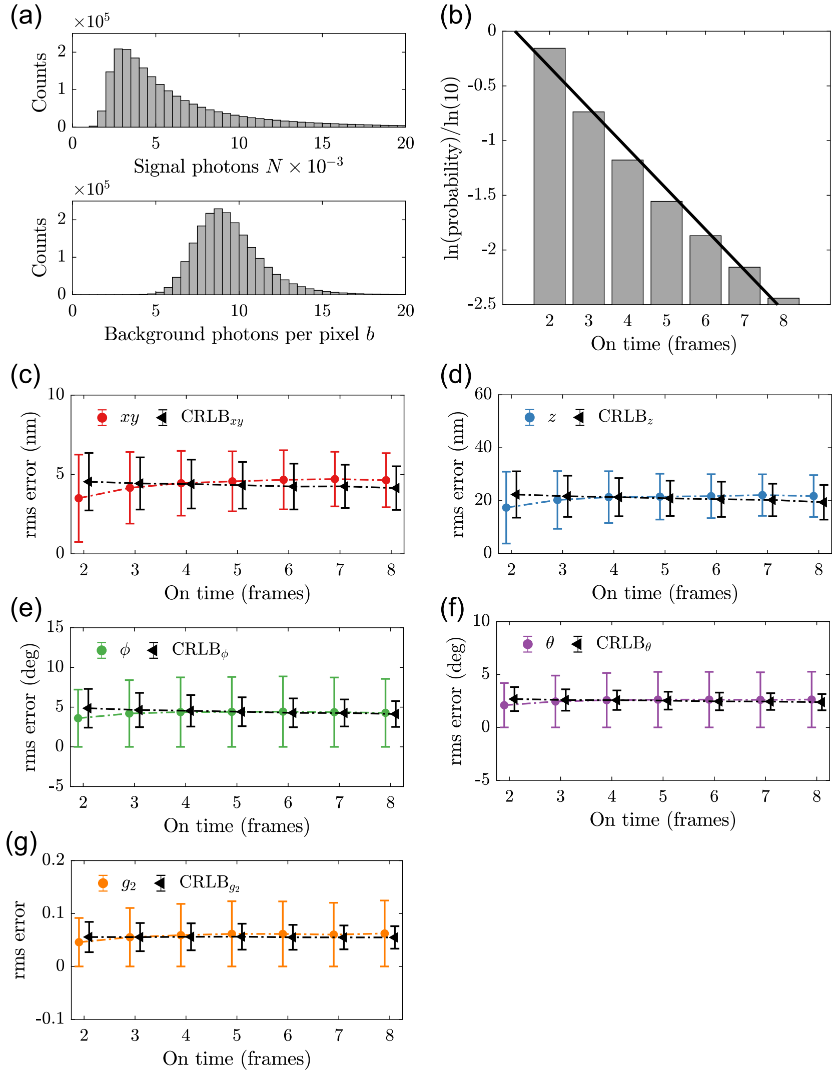

Figure 10: Single-molecule run length analysis in λ-DNA experiment. (a) Distribution of estimated signal photon count N and background photon count per pixel b. (b) Natural-logarithm probability-distribution (gray bars) of subsequent on-time events up to 8 frames for the same emitter. The average on-time over all events is 1.5 frames, whereas the fitted exponential distribution gives an on-time of 1.3 frames (black line). (c) Lateral localization error (root-mean-square value) and CRLB (mean and s.d.) determined from repeated localizations. In the same way, (d) axial localization error, (e) azimuth angle error, (f) polar angle error, and (g) g_2 error as estimated from repeated localizations. The estimated rms error matches well with the estimated CRLB for all parameters and the number of on-time events. The total number of linked on-events is 302,541 with experimental conditions and data analysis as further specified in the Methods.

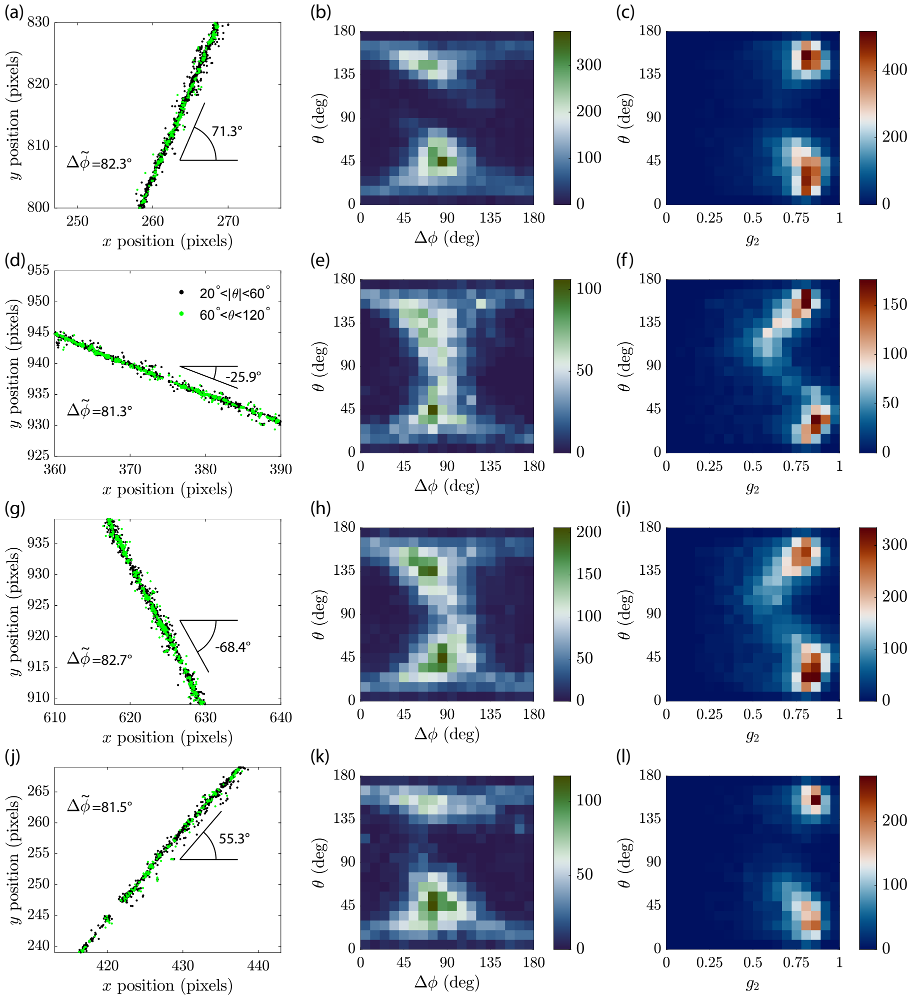

Figure 11: Distributions of various differently oriented λ-DNA strands throughout the FOV of Figure 4(a), illuminated in TIRF conditions with the QWP. (a,d,g,j) Localizations of two different subsets from 4 different strands, in-plane orientations ($60° < \theta < 120°$) in green and out-of-plane orientations ($20° < |\theta| < 60°$) in black. The average DNA orientation and average relative in-plane orientation ($\Delta\widetilde{\phi}$) is indicated with each λ-DNA strand. (b,e,h,k) 2D histogram of the relative in-plane orientation $\Delta\phi$ and the polar angle θ of the respective λ-DNA strands displayed to the left. These histograms show that the orientational parameter distributions are largely independent of the DNA orientation. The variations around $\theta = 90°$ are caused by in-plane orientations that are excited less efficiently as they are perpendicular to the primary polarization direction. (c,f,i,l) 2D histogram of g_2 and θ for the respective λ-DNA strands displayed to the left.

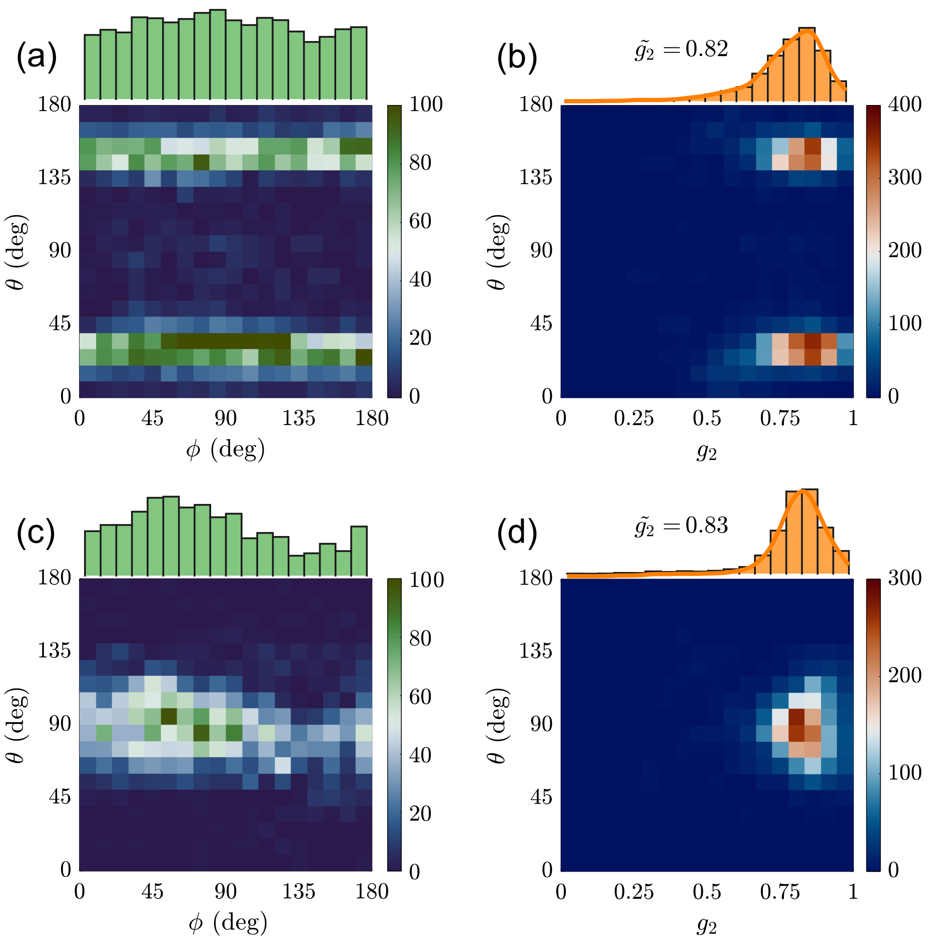

Figure 12: Estimation of orientational parameters on fixed molecules. (a) Bivariate histogram of the azimuth angle (ϕ) versus polar angle (θ) and (b) degree of orientational constraint (g_2) versus polar angle as estimated on single molecules directly spin-coated onto a cover slip under TIRF illumination. The marginal histograms show the ϕ and g_2 distribution, respectively, with the median g_2 coefficient specified in the plot. (c-d) The same as in (a) and (b), but estimated on single molecules embedded in a thin layer of PMMA under non-TIRF epi-illumination. In both these experimental cases with different orientation distributions, there appears to be no correlation between the estimated parameters. The number of single molecules analyzed is 7042 in (a-b) and 4034 in (c-d) with sample preparation and imaging conditions as further specified in the Methods.

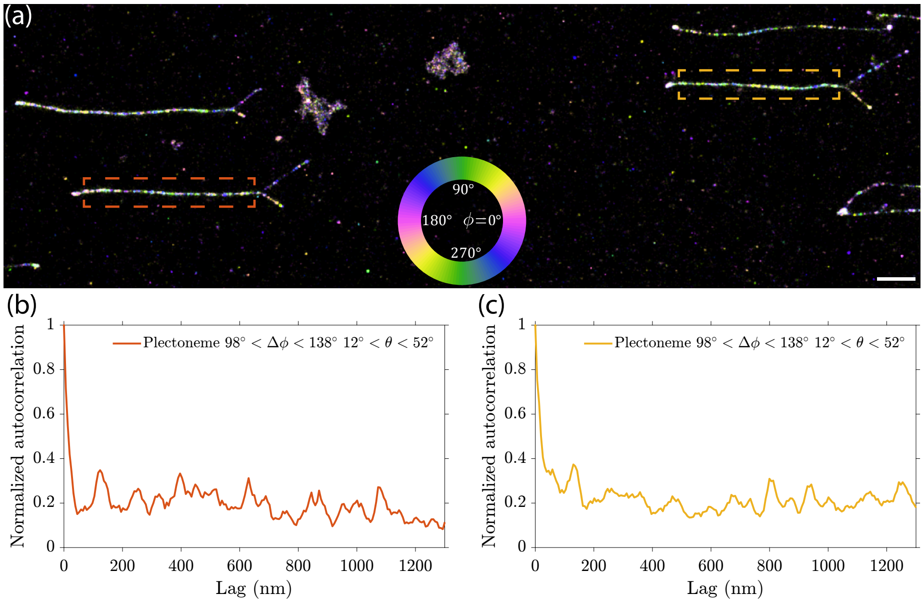

Figure 13: Orientation and spatial correlation of angular subsets in plectonemes. (a) Azimuthal orientation of supercoiled DNA molecules (rotated 90 degrees clockwise to fit figure, scale bar 1 μm). Similar orientations are obtained in different regions of interest and on an additional sample. (b) Autocorrelation of localizations along the plectoneme highlighted in orange indicating a periodicity of $\sim$ 122 nm. (c) Autocorrelation of localizations along the plectoneme highlighted in yellow indicating a periodicity of $\sim$ 130 nm. The autocorrelations are calculated along the x axis with localizations binned in 6.5 nm intervals.

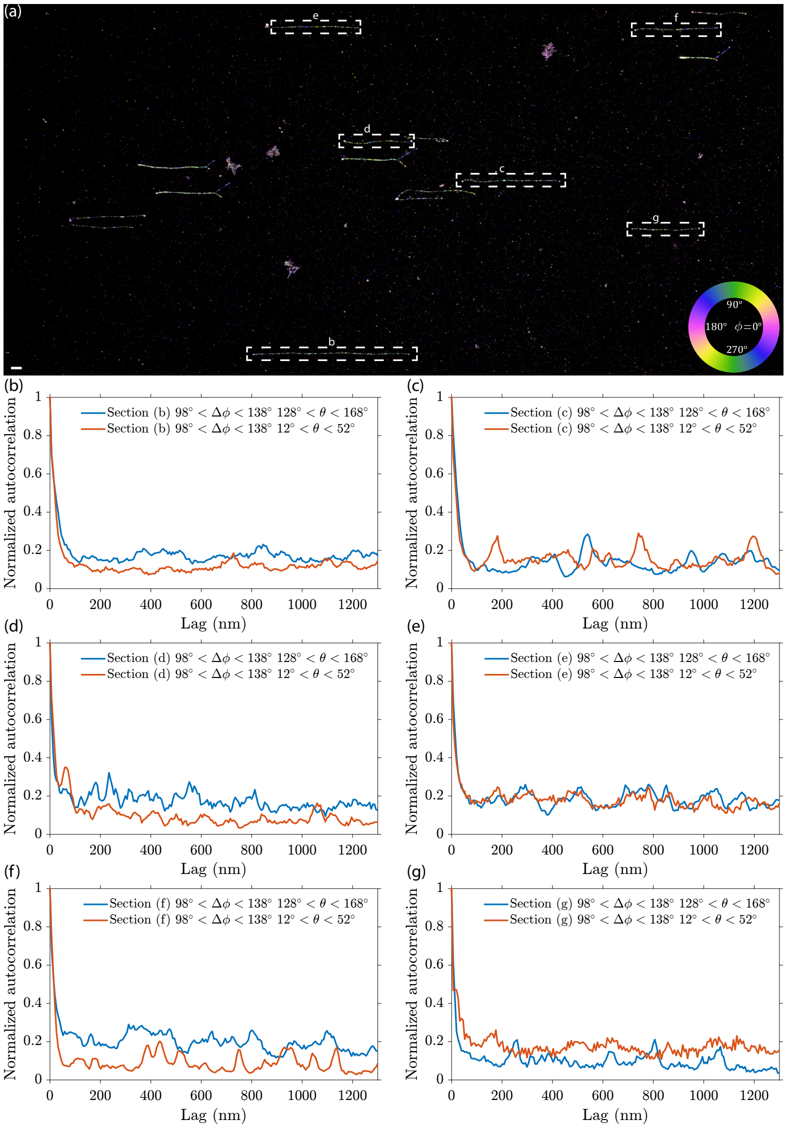

Figure 14: Spatial autocorrelation of sections from torsionally relaxed DNA molecules made in the supercoiled sample preparation, showing that the periodic spatial correlation found on plectonemes is not present on most straight individual strands. This is because these DNA molecules were attached to the coverslip at only one end and hence internal torsion could not be maintained. (a) Average azimuthal orientation (ϕ) of the supercoiled dataset (rotated 90 degrees clockwise to fit figure, scale bar 1 μm) (b)-(g) Autocorrelation of localizations within respective orientation ranges along the DNA axis in 6.5 nm bins from the strands highlighted in (a). Most data show various peaks in the auto correlation but these peaks are not periodic.

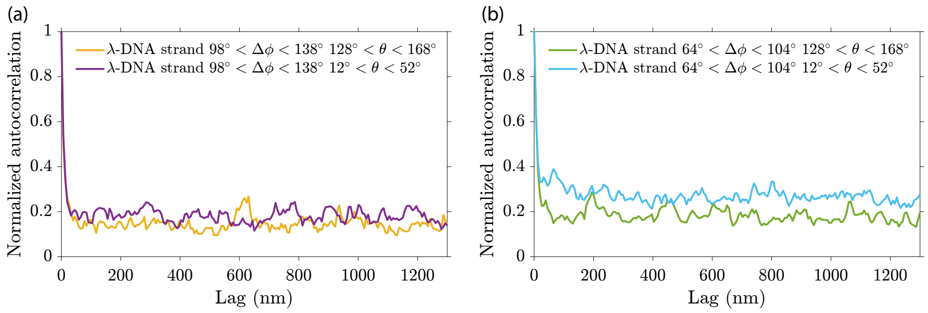

Figure 15: Spatial autocorrelation of the λ-DNA strand highlighted in Figure 4(a), indicating no periodic spatial correlation in the λ-DNA dataset. (a) Autocorrelation of localizations from the λ-DNA strand within respective orientation ranges found from the supercoiled data along the DNA axis in 6.5 nm bins. (b) Autocorrelation of localizations within primary orientation ranges found from the λ-DNA data along the DNA axis in 6.5 nm bins.

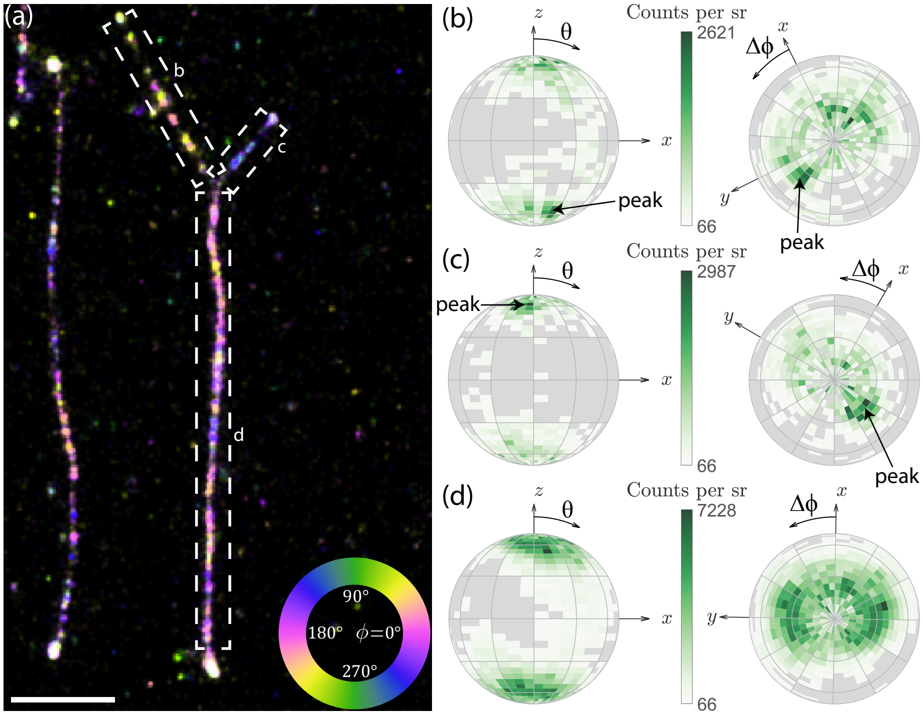

Figure 16: Orientation distribution of the supercoiled sections before and on the plectoneme. (a) Average azimuthal orientation (ϕ) of one of the plectonemes (scale bar 1 μm). (b) Orientation distribution of the top-left supercoiled section before overlap highlighted in (a), with the DNA axis aligned in the x direction. On the left the view is aligned along the y axis, on the right is a top view aligned in the negative z direction. The average orientation around the peak value is $\Delta\phi = 111°$ $\theta = 36°$. (c) Orientation distribution of the top-right supercoiled section before overlap highlighted in (a), with the DNA axis aligned in the x direction. The average orientation around the peak value is $\Delta\phi = -102°$ and $\theta = 29°$. (d) Orientation distribution of the plectoneme with the DNA axis aligned in the x direction highlighted in (a), which appears to show a superposition of the two individual distributions from before the strands intertwined. Each bin spans 5° in the polar direction and 10° in the azimuthal direction, additionally there is a 30° coarse grid. Similar orientations and distributions are obtained on different plectonemes from this sample and on an additional sample.

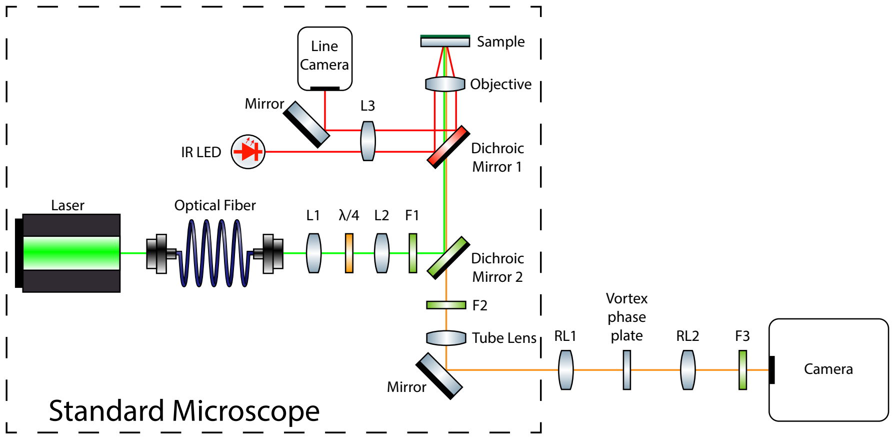

Figure 17: The optical setup, built around a standard fluorescence microscope (Ti-E, Nikon). Lenses RL1 (AC508-100-A-ML, Thorlabs) and RL2 (AC508-100-A-ML, Thorlabs) relay the original image plane to the camera (Zyla 4.2 PLUS, Andor) with no additional magnification resulting in a pixel size of 65 nm in object (sample) space. The vortex phase plate (V-593-10-1, Vortex Photonics) is placed in the Fourier plane between these two lenses. The standard TIRF microscope has a focus lock consisting of an infrared light emitting-diode, offset lens L3, Dichroic Mirror 1 and a line camera (any unspecified components are part of the Nikon Ti-E or its accessories). The excitation laser (Sapphire 561-150 CW, Coherent) is coupled into a fiber and collimated by lens L1 and thereafter focused onto the back focal plane of the objective (CFI Apochromat TIRF 100XC Oil, Nikon) with lens L2. By translating the fiber face the excitation beam angle coming out of the objective can be adjusted to total internal reflection conditions in the sample. The $\lambda/4$ waveplate converts the linearly polarized laser beam to circular polarization, and the excitation spectrum is filtered by F1 (ZET405/488/561/640x, Chroma). Dichroic Mirror 2 (ZT405/488/561/640rpc, Chroma) splits the excitation and emission path and the emission spectrum is further filtered by F2 (ZET405/488/561/640m-TRF, Chroma) and F3 (FF01-609/57-25, Semrock). The tube lens focuses the image from the sample to the front focal plane of lens RL1.

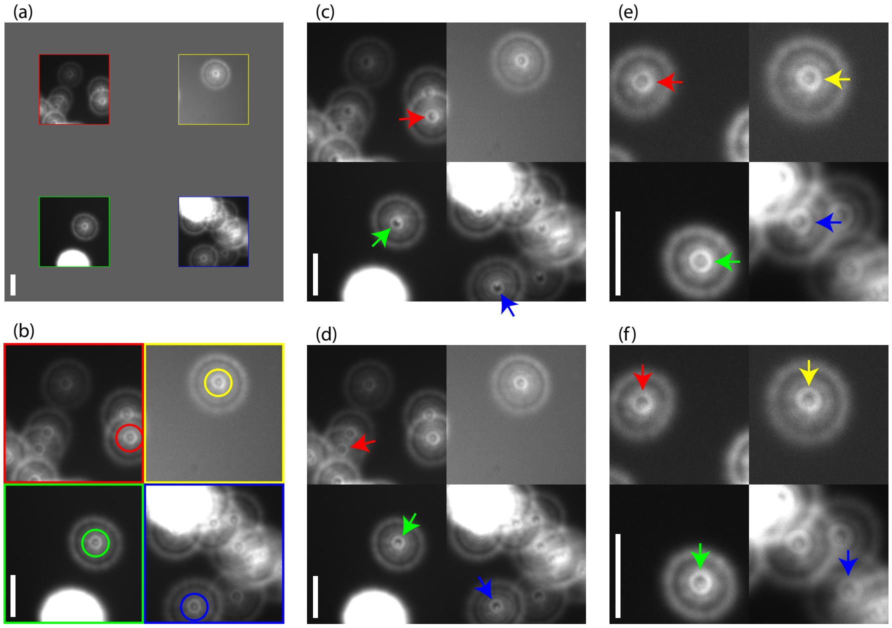

Figure 18: Vortex phase plate alignment. (a) Selected imaged regions of defocused beads on the camera. (b) Zoom in on the four selected regions. The circles highlight the central region where a central peak is surrounded by a bright ring. The bright ring moves as the vortex phase plate is moved, in this image the center of the rings and the peaks coincide and thus the phase plate is properly aligned. (c) The vortex phase plate is too close to the microscope as the rings created by the vortex phase plate are too far radially outward. The arrows indicate the direction in which the rings should be moved. (d) The vortex phase plate is too close to the camera when the rings are too far radially inward. (e) When the vortex phase plate is aligned along the optical axis all spots should look the same throughout the FOV. The position can then be fine-tuned by shifting horizontally. (f) Vortex phase plate slightly misaligned vertically. Scale bars are 10 μm.

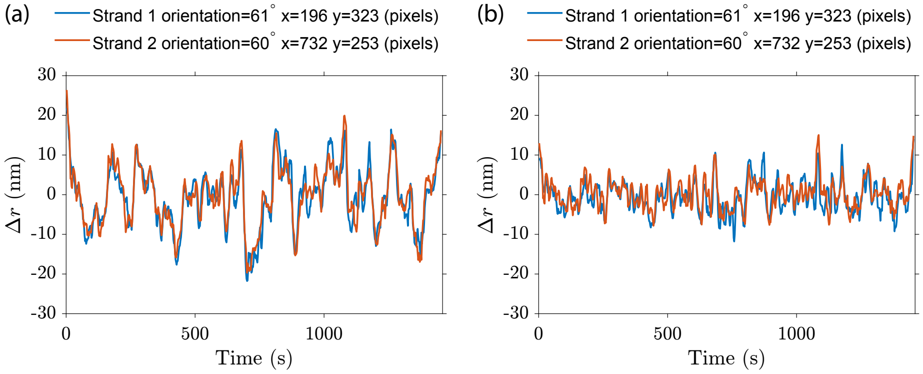

Figure 19: Fine drift correction using straight λ-DNA strands illustrated on two similarly oriented λ-DNA strands at distant positions in the FOV (35 μm apart). (a) Mean deviations from the spline fit to the two DNA strands over time after coarse drift correction. Each data point is the average relative position over 100 frames (10.5 seconds) and shifted in 20 frame steps. The coarse drift correction has corrected $\sim$ 100 nm of slow time scale drift. There is however still a sizeable amount of residual drift as evident by the correlation between the deviations of the two considered λ-DNA strands. The deviations of these two strands have a standard deviation of 7.6 nm over time. The average width of the distribution of localizations around the spline fits of these two λ-DNA strands is 24.5 nm FWHM after coarse drift correction. (b) Mean deviations in 100 frame bins after fine drift correction (as described in data analysis in the Methods section) shows a reduction in the drift amplitude giving a residual standard deviation of 3.7 nm. The fine drift correction reduces the mean width of localizations on these two λ-DNA strands to 18.3 nm FWHM.

References

[1] Mortensen, K. I., Churchman, L. S., Spudich, J. A. & Flyvbjerg, H. Optimized localization analysis for single-molecule tracking and super-resolution microscopy. *Nat. Methods* **7**, 377–381 (2010).

[2] Stallinga, S. Effect of rotational diffusion in an orientational potential well on the point spread function of electric dipole emitters. *J. Opt. Soc. Am. A* **32**, 213 (2015).

[3] Backer, A. S. & Moerner, W. E. Determining the rotational mobility of a single molecule from a single image: a numerical study. *Opt. Express* **23**, 4255 (2015).

[4] Zhang, O. & Lew, M. D. Fundamental limits on measuring the rotational constraint of single molecules using fluorescence microscopy. *Phys. Rev. Lett.* **122**, 198301 (2019).

[5] Chandler, T., Shroff, H., Oldenbourg, R. & La Rivière, P. Spatio-angular fluorescence microscopy I Basic theory. *J. Opt. Soc. Am. A* **36**, 1334 (2019).

[6] Chandler, T., Shroff, H., Oldenbourg, R. & La Rivière, P. Spatio-angular fluorescence microscopy III Constrained angular diffusion, polarized excitation, and high-NA imaging. *J. Opt. Soc. Am. A* **37**, 1465 (2020).

[7] Stallinga, S. & Rieger, B. Accuracy of the Gaussian point spread function model in 2D localization microscopy. *Opt. Express* **18**, 24461 (2010).

[8] Smith, C., Huisman, M., Siemons, M., Grünwald, D. & Stallinga, S. Simultaneous measurement of emission color and 3D position of single molecules. *Opt. Express* **24**, 4996 (2016).

[9] Yan, T., Richardson, C. J., Zhang, M. & Gahlmann, A. Computational correction of spatially variant optical aberrations in 3D single-molecule localization microscopy. *Opt. Express* **27**, 12582 (2019).

[10] Siemons, M. *et al.* Comparing strategies for deep astigmatism-based single-molecule localization microscopy. *Biomed. Opt. Express* **11**, 735 (2020).

[11] Siemons, M., Hulleman, C. N., Thorsen, R. Ø., Smith, C. S. & Stallinga, S. High precision wavefront control in point spread function engineering for single emitter localization. *Opt. Express* **26**, 8397 (2018).

[12] Shack, R. V. & Thompson, K. Influence of alignment errors of a telescope system on its aberration field. In *Optical Alignment I*, vol. 0251, 146–153. International Society for Optics and Photonics (SPIE, 1980).

[13] Tessieres, R. *Analysis for alignment of optical systems.* M.sc. thesis, The University of Arizona (2003).

[14] Zhang, O., Lu, J., Ding, T. & Lew, M. D. Imaging the three-dimensional orientation and rotational mobility of fluorescent emitters using the Tri-spot point spread function. *Appl. Phys. Lett.* **113**, 031103 (2018).

[15] Ding, T., Wu, T., Mazidi, H., Zhang, O. & Lew, M. D. Single-molecule orientation localization microscopy for resolving structural heterogeneities between amyloid fibrils. *Optica* **7**, 602 (2020).

[16] Curcio, V., Alemán-Castañeda, L. A., Brown, T. G., Brasselet, S. & Alonso, M. A. Birefringent Fourier filtering for single molecule coordinate and height super-resolution imaging with dithering and orientation. *Nat. Commun.* **11**, 5307 (2020).
