# Supplementary material for: Simultaneous orientation and 3D localization microscopy with a Vortex point spread function
Source: Nat Commun. 2021 Oct 11;12:5934. doi: 10.1038/s41467-021-26228-5 (PMC8505439; doi:10.1038/s41467-021-26228-5)
Supplement: Supplementary file 3 — Description of Additional Supplementary Files [file 41467_2021_26228_MOESM3_ESM.pdf]

Description of Additional Supplementary Files

Supplementary Movie 1 :

Re-orienting single ATTO 565 molecules in air imaged with the Vortex PSF and TIRF illumination with an exposure time of 900 ms.
