# Supplementary material for: Simultaneous orientation and 3D localization microscopy with a Vortex point spread function
Source: Nat Commun. 2021 Oct 11;12:5934. doi: 10.1038/s41467-021-26228-5 (PMC8505439; doi:10.1038/s41467-021-26228-5)
Supplement: Supplementary file 5 — Reporting Summary [file 41467_2021_26228_MOESM5_ESM.pdf]

nature research

Corresponding author(s): B. Rieger

Last updated by author(s): Aug 22, 2021

Reporting Summary

Nature Research wishes to improve the reproducibility of the work that we publish. This form provides structure for consistency and transparency in reporting. For further information on Nature Research policies, see our Editorial Policies and the Editorial Policy Checklist.

Statistics

For all statistical analyses, confirm that the following items are present in the figure legend, table legend, main text, or Methods section.

n/a	Confirmed	
☐	☒	The exact sample size (*n*) for each experimental group/condition, given as a discrete number and unit of measurement
☒	☐	A statement on whether measurements were taken from distinct samples or whether the same sample was measured repeatedly
☒	☐	The statistical test(s) used AND whether they are one- or two-sided<br>*Only common tests should be described solely by name; describe more complex techniques in the Methods section.*
☒	☐	A description of all covariates tested
☒	☐	A description of any assumptions or corrections, such as tests of normality and adjustment for multiple comparisons
☐	☒	A full description of the statistical parameters including central tendency (e.g. means) or other basic estimates (e.g. regression coefficient) AND variation (e.g. standard deviation) or associated estimates of uncertainty (e.g. confidence intervals)
☒	☐	For null hypothesis testing, the test statistic (e.g. *F*, *t*, *r*) with confidence intervals, effect sizes, degrees of freedom and *P* value noted<br>*Give P values as exact values whenever suitable.*
☒	☐	For Bayesian analysis, information on the choice of priors and Markov chain Monte Carlo settings
☒	☐	For hierarchical and complex designs, identification of the appropriate level for tests and full reporting of outcomes
☒	☐	Estimates of effect sizes (e.g. Cohen's *d*, Pearson's *r*), indicating how they were calculated

Our web collection on statistics for biologists contains articles on many of the points above.

Software and code

Policy information about availability of computer code

Data collection	For microscope control we used NIS-Elements AR (v 4.13.01). For image acquisition we used Andor Solis (v 4.31.30024.0) and for the supercoiled DNA image acquisition we used HCImage (v 4.5.1.3).
Data analysis	MATLAB code for Vortex PSF simulation, fitting and aberration calibration is available on github.com/imphys/vecfitcpu_vortex. This code is tested and used in MATLAB 2018-2020. Additionally the DIPImage toolbox (v 2.9) for MATLAB is requried (diplib.org). Picasso software (v 0.2.8) is used for coarse drift correction (github.com/jungmannlab/picasso). Localization visualization is done using Gaussian blurring from the INSPR toolbox (github.com/HuanglabPurdue/INSPR).

For manuscripts utilizing custom algorithms or software that are central to the research but not yet described in published literature, software must be made available to editors and reviewers. We strongly encourage code deposition in a community repository (e.g. GitHub). See the Nature Research guidelines for submitting code & software for further information.

Data

Policy information about availability of data

All manuscripts must include a data availability statement. This statement should provide the following information, where applicable:

- Accession codes, unique identifiers, or web links for publicly available datasets
- A list of figures that have associated raw data
- A description of any restrictions on data availability

Raw image files and processed localization data is available here (10.4121/c.5136125).

Field-specific reporting

Please select the one below that is the best fit for your research. If you are not sure, read the appropriate sections before making your selection.

☒ Life sciences ☐ Behavioural & social sciences ☐ Ecological, evolutionary & environmental sciences

For a reference copy of the document with all sections, see nature.com/documents/nr-reporting-summary-flat.pdf

Life sciences study design

All studies must disclose on these points even when the disclosure is negative.

Sample size	Sample sizes are determined by the amount of data that meets the filtering criteria specified in the methods. This typically implies that all non-overlapping fluorescent molecules of sufficient signal-to-background ratio were analyzed. Additionally for the z-stacks the fluorescent molecules need to be stable and present in both the dataset with and without the vortex phase plate.
Data exclusions	Supplementary Fig. 6 (b) only shows orientations of single fluorescent molecules present in both the dataset with and without the vortex phase plate (due to bleaching and blinking not all molecules are stable during both acquisitions). Figure 3 highlights 3 reorienting fluorescent single molecules, from Supplementary Movie 1 it is clear many more fluorescent molecules have such reorientation events, however not all of them can be visualized simultaneously in the figure. Figure 4 (a, e, f), fig. 5 (a) and fig. 6 (c) only show a zoomed in subsection of the entire dataset (otherwise the details are too small to visualize).
Replication	The properties of the λ-DNA molecule highlighted in figure 4 are similar to those from other λ-DNA molecules with different orientations (shown in Supplementary figure 11), additionally similar properties have been found in all acquired datasets. For the plectonemic supercoiled DNA similar properties have been found on 3 different plectonemes (Figure 6 and Supplementary figure 13 & 16), in contrast to 6 torsionally relaxed DNA molecules (Supplementary Figure 14). Similar properties have been found in an additional supercoiled DNA dataset.
Randomization	N.A. as samples were not allocated into experimental groups
Blinding	N.A. as there is no group allocation

Reporting for specific materials, systems and methods

We require information from authors about some types of materials, experimental systems and methods used in many studies. Here, indicate whether each material, system or method listed is relevant to your study. If you are not sure if a list item applies to your research, read the appropriate section before selecting a response.

Materials & experimental systems

n/a	Involved in the study	
☒	☐	Antibodies
☒	☐	Eukaryotic cell lines
☒	☐	Palaeontology and archaeology
☒	☐	Animals and other organisms
☒	☐	Human research participants
☒	☐	Clinical data
☒	☐	Dual use research of concern

Methods

n/a	Involved in the study	
☒	☐	ChIP-seq
☒	☐	Flow cytometry
☒	☐	MRI-based neuroimaging
